# Otomycosis Caused by *Aspergillus* Identified by Characteristic Otoscopic Findings

**DOI:** 10.1002/jgf2.70103

**Published:** 2026-02-15

**Authors:** Kento Koda

**Affiliations:** ^1^ Department of Otolaryngology–Head and Neck Surgery The University of Tokyo Hospital Tokyo Japan; ^2^ Department of Otolaryngology Kamagaya General Hospital Chiba Japan

**Keywords:** *aspergillus*, clinical image, external auditory canal, otomycosis, otoscopic findings

An 84‐year‐old woman presented to an otolaryngology clinic with a 3‐week history of right‐sided otalgia and persistent pruritus. She had no fever or otorrhea, and her medical history was notable only for well‐controlled hypertension. She had not used topical antibiotics or steroid‐containing ear drops prior to presentation. Otoscopic examination revealed dense white filamentous material with numerous black spores filling the right external auditory canal, obscuring visualization of the tympanic membrane (Figure 1).

**FIGURE 1:**
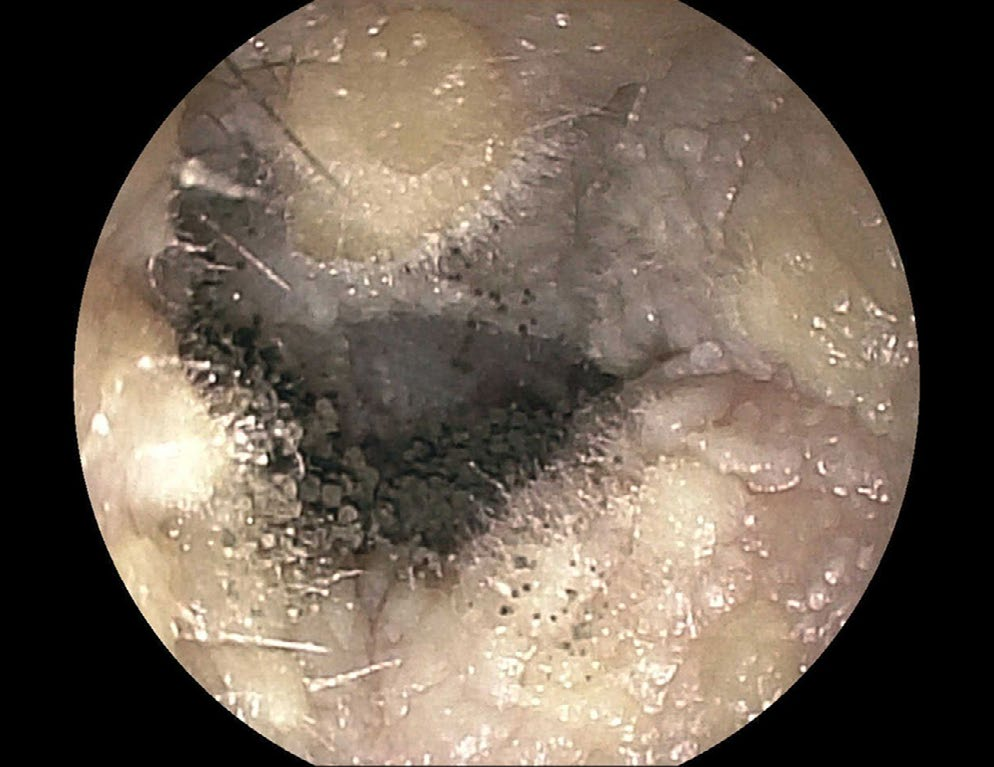
Otoscopic image of the right external auditory canal showing dense white filamentous material with numerous black spores, characteristic of *Aspergillus* otomycosis.

Based on these characteristic otoscopic findings, otomycosis was suspected, and careful mechanical debridement of the external auditory canal was performed as thoroughly as possible. After removal of the fungal mass, mild erythema of the canal skin was observed. The external auditory canal was subsequently irrigated with normal saline, and luliconazole cream was instilled into the canal using a syringe fitted with the outer sheath of a 21‐gauge Surflo catheter. Because the tympanic membrane on the affected side was not clearly visible, the insertion depth of the catheter was determined with reference to the contralateral tympanic membrane depth, with careful attention to avoid tympanic membrane injury.

At the 1‐week follow‐up, the fungal filaments had almost completely resolved; irrigation and topical antifungal treatment were repeated. Complete resolution was confirmed after 2 weeks. The patient was followed for 6 months thereafter, during which no recurrence was observed.

Subsequent fungal culture confirmed infection with *Aspergillus* species. Fungal identification was performed using potassium hydroxide (KOH) direct microscopy and fungal culture on Sabouraud dextrose agar. Antifungal susceptibility testing was not performed because of limitations in the institutional laboratory system.

Otomycosis commonly occurs in humid environments and is associated with external auditory canal trauma or cleaning habits, use of hearing aids or other devices, diabetes mellitus, and immunosuppression. In addition, prior use of topical antibiotics or steroid‐containing ear drops may disrupt the local environment of the external auditory canal and promote fungal overgrowth, potentially prolonging symptoms [1]. The most common causative organisms are *Aspergillus* and *Candida* species [2, 3].

Management of otomycosis primarily consists of topical antifungal therapy combined with ear irrigation and mechanical debridement, and symptoms typically resolve within 1–2 weeks. However, recurrence may occur if predisposing factors persist. *Aspergillus* species often present with characteristic otoscopic findings, including black spores and dense fungal masses [3].

Systemic triazole antifungal agents, such as fluconazole, have limited activity against *Aspergillus* species and are therefore not recommended for uncomplicated otomycosis [2]. In the external auditory canal, cream formulations may be preferable to ointments, as they spread more evenly and are less likely to cause canal obstruction or promote moisture retention.

Early recognition of otoscopic findings suggestive of *Aspergillus* infection facilitates appropriate treatment selection and may contribute to prompt symptom resolution and prevention of recurrence.

## Author Contributions

**Kento Koda:** conceptualization, data curation, formal analysis, supervision, project administration, visualization, writing – original draft, writing – review and editing.

## Funding

The author has nothing to report.

## Ethics Statement

This clinical image was prepared in accordance with the Declaration of Helsinki.

## Consent

Written informed consent was obtained from the patient for publication of this clinical image. Patient anonymity has been preserved.

## Conflicts of Interest

The author declares no conflicts of interest.

## Data Availability

The data that support the findings of this study are available from the corresponding author upon reasonable request.
